# Analgesia by Cryotherapy in Patients with Chronic Pain with Analysis of Pain-Modulating and Pro-Inflammatory Parameters—A Clinical Controlled Pilot Study

**DOI:** 10.3390/jcm14217567

**Published:** 2025-10-25

**Authors:** Henrike Ritter, Ruth Beuermann, Vera Unkelbach, Holger Bang, Eugen Feist

**Affiliations:** 1 Medical School, Otto-von-Guericke University, 39120 Magdeburg, Germany; ritter.henrike@gmail.com; 2 Helios Department of Rheumatology and Clinical Immunology, Vogelsang, 39245 Gommern, Germany; 3 Sebia Labordiagnostische Systeme GmbH, 55129 Mainz, Germany; hbang@sebia.com; 4 Experimental Rheumatology, Otto-von-Guericke University, 39120 Magdeburg, Germany

**Keywords:** whole-body cryotherapy, chronic pain, fibromyalgia syndrome, substance P, calprotectin, calcitonin gene-related peptide, β-nerve growth factor, ELISA

## Abstract

**Background/Objectives**: Whole-body cryotherapy (WBC) is increasingly utilized as a physical modality for managing chronic pain, although its mechanism of action remains incompletely understood. This study evaluated whether WBC influences serum levels of substance P, calprotectin, β-nerve growth factor (β-NGF), and calcitonin gene-related peptide (CGRP), which are implicated in pain modulation. **Methods**: Serum samples from 61 participants—37 undergoing WBC and 24 not receiving WBC—were collected at the start and end of a multimodal inpatient pain treatment program. Pain intensity was assessed using a numerical rating scale (NRS). Biomarker concentrations were measured by enzyme-linked immunosorbent assay (ELISA). **Results**: Both groups reported an average significant pain reduction of more than 1.39 points on the NRS. Of the biomarkers analyzed, only calprotectin showed a statistically significant reduction in the overall cohort (*p* = 0.007) and in the WBC subgroup (*p* = 0.032). Among patients who did not experience significant pain reduction, those in the WBC group exhibited a greater decline in calprotectin compared to controls (*p* = 0.042), especially among those without medication changes (*p* = 0.016). No significant differences were detected for the other serum parameters. **Conclusions**: The analgesic effects of WBC could not be attributed to changes in the neuromodulatory peptides measured. However, the significant reduction in calprotectin suggests a potential anti-inflammatory effect of WBC on the innate immune response.

## 1. Introduction

Pain is a widespread phenomenon, and its underlying mechanisms are not fully understood—particularly in chronic pain conditions. Managing pain is central to the treatment of many diseases and remains especially challenging in rheumatic disorders [1]. Accordingly, effective pain relief and maintenance of function are primary goals of treatment strategies.

At a physiological level, pain is defined as “an unpleasant subjective experience typically caused by, or comparable to, actual or potential tissue injury” [2]. Nociception refers to the sensory processes that detect and process noxious stimuli, relying on specialized receptors in the nervous system. One important group of such receptors is the transient receptor potential (TRP) ion channels, which play a critical role in pain sensation. TRP channels are multimodal sensors that can be activated by chemical stimuli (e.g., capsaicin) as well as by thermal stimuli (heat or cold). In injured or inflamed tissues, the expression of certain TRP channels (particularly on unmyelinated C-fibers) is upregulated, leading to a sensitization response [3]. Furthermore, the activity of TRP channels can be enhanced by β-nerve growth factor (β-NGF), which induces the release of the neuropeptides calcitonin gene-related peptide (CGRP) and substance P. These neuropeptides trigger an immune response that contributes to neurogenic inflammation. Among the TRP channels, the vanilloid subtype (TRPV1) is especially noteworthy: it mediates normal nociceptive responses to heat and is a key driver of pathologic pain phenomena such as thermal hyperalgesia, mechanical allodynia, and spontaneous pain during inflammation [4].

Another mediator linking the immune system and pain signaling is calprotectin, an acute-phase protein that reflects neutrophil activity [5]. Neutrophils release calprotectin upon activation, and it can also be released passively during neutrophil cell death [6]. Calprotectin signals through the Toll-like receptor 4 (TLR4), which has been implicated in pain perception because it is expressed by afferent neurons [7].

Importantly, when pain becomes chronic, it may lose its initial protective purpose and evolve into an independent disease. Chronic pain is defined as pain in one or more anatomical regions that persists or recurs for at least 3 months, accompanied by significant distress or functional limitation that cannot be adequately explained by another diagnosis [8]. The International Classification of Diseases, 11th Revision (ICD-11), introduced the concept of chronic primary pain to categorize such conditions. This category includes chronic widespread pain—exemplified by fibromyalgia syndrome (FMS), which often presents additional psychosomatic symptoms as well as conditions such as complex regional pain syndrome (CRPS), chronic primary headache or orofacial pain, visceral pain, and chronic musculoskeletal pain. However, the molecular mechanisms underlying the development of chronic pain remain largely unclear. To date, only altered gene expression of certain neurotrophins has been observed in regenerating neurons after nerve injury [9].

Consequently, managing chronic pain is challenging, as it often entails a protracted course with multiple unsuccessful treatments in a patient’s history. Best-practice guidelines advocate interdisciplinary, multimodal pain management that combines medical (including pharmacological) interventions with physical therapy and exercise, psychoeducation, psychological therapy, and the treatment of coexisting physical or psychological disorders [10].

Among these therapeutic modalities, exposure to cold stands out for its potent analgesic effect in chronic pain management [11]. Whole-body cryotherapy (WBC) has emerged as a component of modern multimodal pain therapy [12]. This physical modality has shown promise in achieving sustained pain reduction, even in conditions that are otherwise refractory to treatment. However, the exact mechanism of WBC’s analgesic effect remains unclear, although it is thought to modulate neuro-immune and neuromodulatory pathways involved in pain.

WBC was first used therapeutically in the field of inflammatory rheumatic diseases in Japan in 1980, and shortly thereafter in Germany [13]. During therapy patients pass through several connected cryotherapy chambers that consecutively get colder with temperatures as low as −110 °C. In contrast, partial cryotherapy (PBC) uses a single cryotherapy chamber, leaving the patient’s head untouched. The two systems also differ in the way the cold is generated. While in WBC, compressors prevent direct contact with the cooling liquid nitrogen, in PBC, cooling occurs through direct contact with evaporating nitrogen [14].

In patients with rheumatoid arthritis (RA), WBC has been shown to produce improvements in pain and disease activity comparable to conventional physiotherapy, with the added benefit of markedly reducing fatigue symptoms [15]. Moreover, when compared to standard care, WBC leads to greater pain relief that can persist for up to 12 weeks post-treatment [16]. Even in chronic back pain, notable pain reduction has been observed after as few as four WBC sessions [17].

On a physiological level, the extreme cold exposure during WBC acutely activates the sympathetic nervous system, causing a surge of catecholamines (adrenaline and noradrenaline). Notably, in one study, healthy women undergoing WBC exhibited a two- to threefold increase in circulating noradrenaline after 12 weeks of regular treatment, accompanied by significantly lower levels of adrenocorticotropic hormone (ACTH) and cortisol [18]. WBC also induces an anti-inflammatory cytokine profile: levels of interleukin-10 (IL-10) rise, whereas pro-inflammatory mediators such as IL-6 are suppressed [19]. In particular, adrenaline and noradrenaline surges are thought to stimulate IL-10 production via a cyclic adenosine monophosphate (cAMP)-mediated activation of monocytes [20].

The facts presented above suggest that chronic pain involves an altered usage of neuro-modulatory substances. Whole-body cryotherapy has proven to be an effective means of pain relief in clinical practice. Current data also demonstrate an influence of WBC on inflammatory mediators. The pain modulators β-NGF, substance P, and CGRP have been identified in studies as factors in pain mediation and chronicity. Calprotectin, an acute-phase protein, also shows potential for pain-modulating effects.

Accordingly, the aim of this study was to determine whether WBC induces measurable changes in the serum levels of these pain-modulating markers—calprotectin, substance P, CGRP, and β-NGF—thereby supporting a modulatory effect of WBC on pain pathways. In addition, we evaluated patients’ subjective pain levels, hypothesizing that WBC would lead to a significant reduction in perceived pain.

## 2. Materials and Methods

### 2.1. Recruitment

The experimental study was planned and conducted at the Helios Vogelsang Gommern Hospital, Germany, with approval from Ethics Committee of Otto-von-Guericke-Universität Magdeburg, Germany, (13/21, 20 April 2021). Serum samples were taken from patients with chronic pain, and current pain activity was assessed using the NRS (scale 0–10) at the beginning and end of treatment. All subjects were recruited as part of their interdisciplinary complex pain therapy treatment. This treatment comprises a variety of therapies offered by the Helios Vogelsang Gommern Hospital. Aside from medication adjustment, the patients have access to a wide spectrum of specialized physical therapy, acupuncture, local anesthesia, ergotherapy, nutrition advice, psychological–psychotherapeutic procedures and more. Only consenting patients with a stay of at least 10 days and chronic pain symptoms were included, since these patients underwent the whole treatment protocol that is standardized for German healthcare and adjusted to patient needs. Patients were recruited over a period of 1 year while visiting the hospital for complex treatment.

### 2.2. Population

A total of 64 patients agreed to participate in the study. Of these, 3 patients had to be excluded due to a shorter stay (*n* = 1) or a lack of follow-up blood sample (*n* = 2). The measurements were performed using sera from 61 subjects. Patients were characterized upon admission according to their primary diagnosis, comorbidities and adjustments in medication (Appendix A) and divided into a control group and a test group, which would undergo the WBC. Possible flares in pain or exacerbations of disease were not screened. Assigning patients to treatment and control groups was carried out by the medical personnel following medical reasons and personal patient preferences. Both groups were treated according to the interdisciplinary complex pain therapy, but only the test group had additional WBC.

### 2.3. Execution of Cryotherapy

The patients in the test group visited the in-house cryochamber at the Helios Vogelsang Gommern Specialist Hospital at least once a day as part of their complex pain therapy treatment. The patients could choose to visit the cryochamber up to 2 times daily. The number of usages was not recorded. The model, manufactured by Zimmer Medizin Systeme GmbH (Neu-Ulm, Germany), was developed in accordance with the requirements of IEC 60601-1 and bears CE marking 0123 in accordance with the EG Medical Device Directive 93/42/EWG. Regular maintenance and validation follow the in-house quality management protocol.

The chamber comprises three individual rooms, the first of which, at approximately −16 °C, and the second at approximately −60 °C, serve for acclimatization and start the cooling process. Patients spend approximately 20–30 s in the first two chambers before moving on to the next. The majority of the cryotherapy takes place in the third, at approximately −110 °C up to 3 min. Patients are encouraged to make full use of the three-minute stay, but not to exceed this time due to possible side effects such as skin damage caused by cold. During the therapy session patients wear swimwear and gloves, a headband and a face mask to maximize the cold exposure to the body while avoiding cold-induced damage to the extremities. Duration of acclimatization and stay in the third chamber were not registered separately for each patient.

### 2.4. Laboratory Testing

The serum samples to be tested were collected by the doctoral student or nursing staff upon admission and on the last day of the patient’s 10-day stay from 3 November 2021 to 23 November 2022. A total of 122 sera were collected. The samples were centrifuged at 3000 rpm for 10 min [21] and stored at −20 °C. The respective parameters were measured using various ELISA kits with duplicate determinations. Dosing pipettes from Eppendorf (Hamburg, Germany), which are maintained according to quality assurance, were used. A Euroimmun (Lübeck, Germany) device with a three-run wash program was used to wash the microtiter plates. All plates were read using a Tecan Sunrise (Männedorf, Switzerland) photometer at 450 nm.

A non-diagnostic test kit from Enzo Life Sciences (Long Island, NY, USA) was used for the quantitative determination of substance P in serum. It uses a competitive principle of action, in which diluted samples are added to IgG antigen-coated microwell plates together with an alkaline phosphatase-conjugated substance P solution. There is an indirect proportionality between substance P in serum and the measured optical density of the microwell plate [22]. The non-diagnostic ELISA kit from Biomatik (Kitchener, ON, Canada) for measuring CGRP is also based on the competitive ELISA principle. The microwell plate coated with monoclonal CGRP antibodies is simultaneously filled with sample or standard and biotin-labeled CGRP.

A non-diagnostic test from Orgentec (Mainz, Germany) was used for the measurement of calprotectin. According to the manufacturer, the cutoff value for this test kit is 5.3 µg/mL. The kit uses the indirect ELISA principle. The non-diagnostic test used by R&D Systems (Minneapolis, MN, USA) to measure β-NGF is also based on the indirect ELISA principle.

Microsoft Excel was used to record the raw data and patient characteristics, as well as to determine mean values, standard deviations, and medians.

The optical densities measured by the photometer were recorded using the MikroWin 2010 program and assigned to the respective positions on the microtiter plate. For calprotectin and β-NGF, the concentrations were calculated using the standard curve. The plates were laid out according to the respective test instructions. This allowed the concentration to be determined directly after back-calculating the dilution. Using Microsoft Excel, the duplicate determinations were averaged and assigned to the respective subjects or test groups.

The MicroWin 2010 program only allowed directly proportional relationships when programming the templates. Therefore, separate concentration calculations were required for substance P and CGRP. For this purpose, the optical density was plotted against the logarithm of the standard concentration in Microsoft Excel, and a regression line with a functional equation was created. The functional equation was used to determine the sample concentration. The logarithm and dilutions were then calculated accordingly.

### 2.5. Pain Assessment

The NRS was used to assess pain intensity at the beginning and end of the subjects’ stay. This is an established method for the subjective assessment of pain by adult patients [23]. A unidimensional scale with 11 points is used. 0 corresponds to “no pain” and 10 to the “worst imaginable pain” [24]. The NRS was administered verbally during admission and shortly before discharge. Patients were not given any information about their previous pain score and were asked to rate their average pain over the past 24 h. The NRS was chosen as the method of choice since no visual aid or survey form is necessary, and all patients were verbally and consciously able to provide a sufficient and quick self-assessment.

The minimum for a clinically significant difference in NRS scores was set at 1.39 ± 1.05 with a 95% confidence interval. In a cohort of 354 patients in emergency care, this value was established as the threshold for a significant change in pain and thus as an indicator of effective treatment, with no correlation found with gender or pain etiology [25]. In studies assessing chronic pain, a reduction of 1 unit on the NRS can be considered a slight improvement. A reduction of 1.74 units on the NRS can be considered a good to excellent pain reduction [26].

### 2.6. Statistical Analysis

To determine whether there was a significant change between the initial and final values of the respective biomarkers, the Wilcoxon test, a non-parametric rank test, was used to compare two matched samples, as a normal distribution of the values could not be assumed. The Mann–Whitney U test, a non-parametric test to test value distribution between groups, was used to analyze whether the values differed significantly between the groups. The calculations were conducted with the SPSS program (IBM, Armonk, NY, USA, version 29). The asymptotic significance (two-sided test) was set at *p* = 0.05. A power calculation a priori could not be performed since no comparable data was available.

Given that FMS was well represented in our cohort, we also performed a subgroup analysis for patients with FMS. Of the 61 participants, 32 had FMS as their primary diagnosis (21 in the WBC group and 11 in the control group), and 29 had other chronic pain conditions (16 in WBC, 13 in control). Finally, the potential influence of any changes in pain medication during the study period was considered.

### 2.7. Writing

The results were written as a monograph and later adapted for publishing. After a first draft the GenAI Chat GPT 4.5 was used to enhance the quality of phrasing and grammar. The content was reviewed thoroughly and adapted if necessary. 

## 3. Results

### 3.1. Numerical Rating Scale

**Figure 1:**
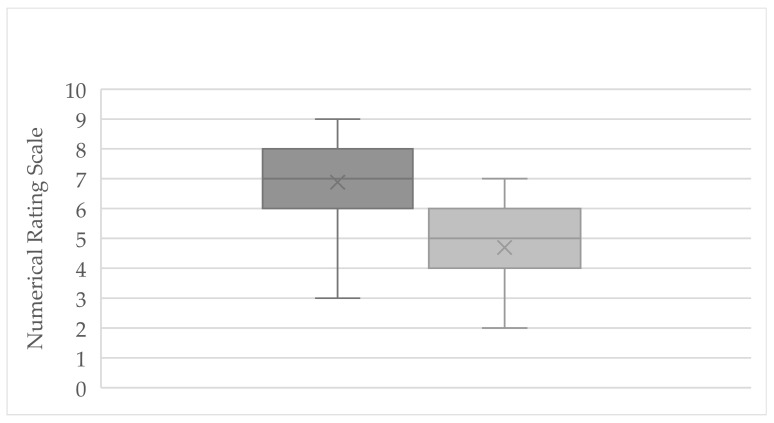
Boxplot of Pain intensity (NRS) in the entire study cohort (*n* = 61) at baseline (dark gray) and post-treatment (light gray), shown as a box-and-whisker plot. The symbol “X” denotes the mean; each box spans from the first to third quartile, with the median represented by the center line; whiskers extend to the minimum and maximum observed values.

At the start of treatment, the entire cohort (*n* = 61) reported an average pain intensity of 6.88 (±1.31) on the NRS (range: 3–8.5). By the end of the observation period, the mean pain score had decreased to 4.70 (±1.25) (range: 2–7), indicating overall pain relief (Figure 1). This corresponds to an average pain reduction of 2.18 points on the NRS (±1.29), with individual changes ranging from −0.5 (minimal improvement) to −4.0 (marked improvement). Three subjects reported no change in pain, and one subject recorded a slight increase of 1 point. Notably, a reduction of ≥1.39 points on the NRS (the predefined threshold for a clinically significant change) was achieved by 45 patients, comprising 73.8% of the cohort.

**Figure 2:**
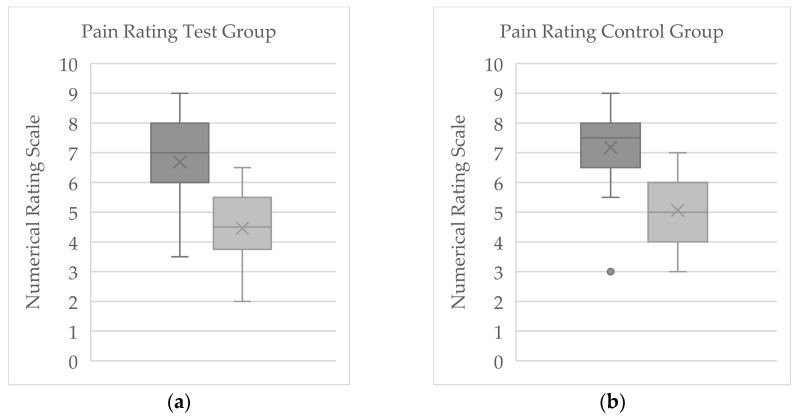
Boxplot of Pain scores (NRS) in the cryotherapy group (*n* = 37) and control group (*n* = 24) at the beginning (dark gray) and end (light gray) of therapy, shown as boxplots. “X” marks the mean; boxes represent the interquartile range with the median as the center line; whiskers indicate the minimum and maximum values. Panel (**a**) displays results for the WBC (cryotherapy) group, and panel (**b**) displays results for the control group.

In the cryotherapy (WBC) group (*n* = 37), the mean baseline NRS pain score was 6.69 (±1.31), with scores ranging from 3.5 to 8.5. By the end of therapy, the mean pain score in this group had fallen to 4.46 (±1.23) (range: 2.5–6.5; Figure 2a). The average within-group change for the WBC group was −2.23 (±1.28) points, with individual pain level changes between −0.5 and −4.0. Two patients (5.4%) in this group reported no change in pain over the treatment period. Using the 1.39-point criterion for clinical significance, 27 out of 37 patients in the cryotherapy group (≈73%) experienced a meaningful reduction in pain. Among those who met this threshold, the mean NRS change was −2.78 (±1.02), indicating a substantial improvement in pain intensity in that subset.

In the control group (*n* = 24) that did not receive cryotherapy, the mean pain score was 7.18 (±1.29) at baseline (range: 3–8.5) and 5.06 (±1.20) at the end of therapy (range: 3–7; Figure 2b). This reflects an average pain decrease of 2.19 points (±1.36) in the control group, with individual changes ranging from −1 to −5 on the NRS. One participant in the control group reported no change in pain, and another reported a slight worsening of pain by 1 point. By the conclusion of therapy, 18 of 24 control patients (75%) achieved a pain reduction of at least 1.39 NRS points, indicating a clinically significant improvement. For these responders in the control group, the mean pain score change was −2.65 (±0.98).

### 3.2. Evaluation of Serum Parameters

#### 3.2.1. Evaluation of Serum Parameters for Significant Change

**Figure 3:**
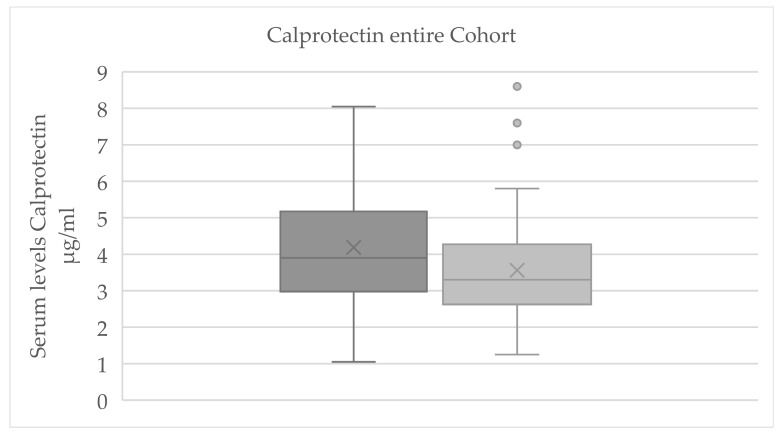
Boxplot of Serum calprotectin concentrations for the entire cohort at baseline (dark gray) and after treatment (light gray) presented as box-and-whisker plots. “X” indicates the mean; boxes show the interquartile range (25th–75th percentiles) with the median as the center line; whiskers represent the minimum and maximum values, and outliers are marked with dots.

For the total cohort, a significant reduction in serum calprotectin levels was observed from baseline to final measurement (*p* = 0.007; Figure 3). A statistically significant change was also detected for β-NGF (*p* = 0.026; see Table 1); however, only 12 out of 61 samples had β-NGF values above the assay’s detection threshold, rendering this result unreliable. This β-NGF finding should therefore be interpreted with caution and was not emphasized in further analyses.

**Figure 4:**
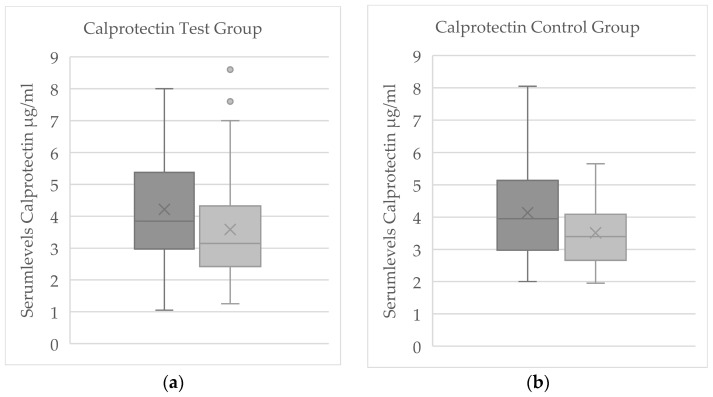
Boxplot of Serum calprotectin concentrations at baseline (dark gray) and after treatment (light gray) presented as box-and-whisker plots. “X” indicates the mean; boxes show the interquartile range (25th–75th percentiles) with the median as the center line; whiskers represent the minimum and maximum values, and outliers are marked with dots. Panel (**a**) corresponds to the cryotherapy group (*n* = 37), and panel (**b**) corresponds to the control group (*n* = 24).

In the control group, none of the reliably measured biomarkers showed a significant pre–post change. The only nominally significant shift in the control group was a decrease in β-NGF, but given the limited number of measurable β-NGF samples and their variability, this apparent change was not deemed meaningful. By contrast, in the cryotherapy test group, calprotectin exhibited a clear within-group decline from baseline to final (*p* = 0.032; Figure 4). No other serum proteins (substance P, β-NGF, or CGRP) showed significant pre–post changes in the WBC group.

#### 3.2.2. Comparison of Serum Levels Between Test and Control Groups

When comparing the cryotherapy and control groups directly, no significant differences were found in the magnitude of change in any serum biomarker between the two groups. In particular, the mean changes in substance P (*p* = 0.988), CGRP (*p* = 0.417), and calprotectin (*p* = 0.982) were statistically equivalent in patients who underwent WBC versus those who did not (Mann–Whitney U tests for between-group differences in change scores).

Given the high proportion of fibromyalgia syndrome (FMS) patients in our sample, we performed a subgroup analysis based on diagnosis. In both the FMS subgroup and the non-FMS subgroup, there were no significant differences between the cryotherapy and control arms in the changes observed for any of the measured serum parameters.

To explore whether the extent of pain relief was associated with biomarker changes, we stratified patients by their pain reduction. Participants were categorized as having either a clinically significant pain improvement (≥1.39 NRS points) or a lesser/no improvement (<1.39 points). Interestingly, among patients who did not reach the 1.39-point pain improvement threshold, the reduction in calprotectin was significantly greater in the WBC group compared to the control group (*p* = 0.042). In contrast, for patients who achieved at least a 1.39-point improvement in pain, there were no significant between-group differences in any serum marker changes.

Finally, to minimize the potential confounding effect of analgesic medication changes, we repeated the analysis for the subset of patients who had no adjustments in their pain medication during the study. In this medication-stable subset, none of the biomarkers showed a significant difference between the cryotherapy and control groups when analyzed as a whole. However, even within this medication-stable subgroup, patients who did not attain a ≥1.39 NRS pain reduction continued to show a markedly larger decrease in calprotectin in the cryotherapy group than in the control group (*p* = 0.016).

## 4. Discussion

In our study we achieved a significant pain reduction for the majority of the participants who received multidisciplinary treatment. WBC, as part of multimodal pain therapy, showed no additional notable decrease in self-reported pain in the test group. In our study setting, the analgesic effect cannot safely be attributed to WBC. It should also be noted that WBC has relatively few side effects [27]. Further investigations without additional treatments might show results that concur with the hypothesis and clinical consensus that WBC shows analgesic effects, but the required study design could not be implemented in the daily hospital routine. The interventions aside from WBC might enhance or subdue analgesic effects, but this could only be explored further in a non-clinical setting with testing singular therapies in a strict treatment protocol.

Of the serum biomarkers analyzed, only calprotectin changed significantly with treatment: its concentration declined in the overall cohort (*p* = 0.007) and specifically in the WBC group (*p* = 0.032), whereas none of the other mediators (substance P, β-NGF, or CGRP) showed a significant alteration in either group. Notably, among patients who did not experience significant pain improvement, calprotectin levels declined more in the WBC group than in the control group (*p* = 0.042), a difference that persisted even when patients with medication adjustments were excluded (*p* = 0.061). This finding reinforces the observation that whole-body cryotherapy may exert an anti-inflammatory effect (as reflected by calprotectin reduction) even in those patients who report minimal subjective pain improvement.

Calprotectin is an acute-phase protein released during inflammation, suggesting that WBC may exert an anti-inflammatory effect. Supporting this interpretation, WBC has been shown to reduce tumor necrosis factor-α (TNF-α) levels in rheumatoid arthritis patients [15], and TNF-α in turn promotes the release of calprotectin [28]. Thus, WBC might attenuate inflammatory cascades by interrupting this TNF-α–calprotectin pathway.

Since we consider our work to be a pilot study, it might yield information for further researchers. Assuming the changes in calprotectin to be of the greatest interest, using our data suggests a sample size of at least 140 patients to achieve a power of 80%.

For further studies it has to be taken into consideration that all patients also received other concurrent treatments as part of the multimodal program, which could have influenced our results. For example, physical exercise included in the regimen has been shown to lower serum calprotectin levels in RA [29]. Furthermore, we did not control for external inflammatory factors such as infections or injuries, which could have contributed to the observed calprotectin reduction independent of cryotherapy [30].

Stratifying outcomes by primary diagnosis (e.g., fibromyalgia syndrome (FMS) versus other chronic pain conditions) yielded no additional insights, indicating that WBC did not have a distinct impact on the measured biomarkers in FMS patients. Likewise, changes in patients’ medication regimens had no discernible influence on these biomarker levels in either the overall cohort or the FMS subgroup.

The biomarkers we examined (substance P, CGRP, β-NGF, and calprotectin) are typically elevated during acute nociceptive or inflammatory responses, whereas our patient cohort had long-established chronic pain. These mediators may fluctuate in the early stages or during the transition from acute to chronic pain, but by the time pain becomes chronic, such changes could have plateaued. In fact, we observed no significant fluctuations in substance P, CGRP, or β-NGF in our chronic pain patients. Nevertheless, WBC might still provide clear pain relief, consistent with the clinical consensus that it is a valuable component of chronic pain and central sensitization treatment [31].

However, the lack of significant changes in most serum markers may also reflect methodological limitations. For instance, the WBC regimen (session intensity and total duration) might have been insufficient to induce measurable neurobiological changes in chronic pain patients. Alternatively, WBC’s analgesic effects could involve pathways or mediators not captured by our serum measurements. To investigate these possibilities, future studies should examine central neurobiological changes. For example, analyzing cerebrospinal fluid (CSF) might be more informative, as central sensitization processes could be evident in CSF even if absent in peripheral blood. Indeed, evidence for a central mechanism comes from observations that WBC improved well-being and reduced oxidative stress in patients with multiple sclerosis, a central nervous system disorder [32].

Subsequent research, perhaps a larger randomized controlled trial isolating WBC’s contribution to pain relief and influence on biomarkers such as calprotectin, can build on these insights.

## 5. Conclusions

Although this study cannot show a notable superior analgesic effect of WBC compared to multimodal pain treatment, it can be stated that this therapeutic option is readily used by patients and is viewed positively.

What has been shown is a significant reduction in calprotectin through WBC and therefore an influence on the innate immune system by decreasing an acute phase protein. Hence, an anti-inflammatory effect can be attributed to WBC.

The current study has set the stage by indicating that something measurable changes in the blood with WBC, which is a significant step beyond simply knowing that patients feel better.

While not a definitive answer on its own, this work pushes the field forward by illuminating a new facet of WBC action.

## Figures and Tables

**Table 1:** Summary of serum biomarker levels in the total cohort (*n* = 61) at baseline (a) and at end of study (b). For each time point, the mean ± standard deviation (SD) and median with 95% confidence intervals (CI) are given. The Z-statistic and *p*-value from paired Wilcoxon tests indicate the significance of pre- to post-treatment changes for each parameter.

Parameter	Baseline Mean ± SD Median [95% CI]	End of Study Mean ± SD Median [95% CI]	Z	*p*
Substance P	3329.3 ± 1075.3 ^2^	3273.9 ± 1209.4 ^2^	−1.267	0.205
	3321.1 [3123.5; 3604] ^2^	3188 [2881.6; 3626.8] ^2^		
Calprotectin	4.2 ± 1.7 ^3^	3.6 ± 1.4 ^3^	−2.713	*0.007*
	3.9 [3.25; 4.3] ^3^	3.3 [2.95; 3.8] ^3^		
β-NGF	140.5 ± 693.3 ^2^	121.9 ± 572.9 ^2^	−2.223	*0.026* ^1^
	0 [0; 0] ^2^	0 [0; 0] ^2^		
CGRP	1424.7 ± 1654.9 ^2^	1383.6 ± 1593.7 ^2^	−0.664	0.506
	621.8 [494.1; 820.9] ^2^	594.8 [521.1; 774.9] ^2^		

^1^ The ELISA test for β-NGF yielded stable results on the calibration curve. However, only 12 of the 61 sera measured β-NGF at a 1:5 dilution. Due to the few and unreliable reproducible values, the evaluation using Wilcoxon tests should be viewed with caution, and the results were not included in further analysis. ^2^ Values given in pg/mL. ^3^ Values given in µg/mL. Significant results in italics.

## Data Availability

Data is unavailable due to privacy restrictions.

## Abbreviations

The following abbreviations are used in this manuscript:

β-NGF	β-nerve growth factor
ACTH	Adrenocorticotropic hormone
cAMP	Cyclic adenosine monophosphate
CGRP	Calcitonine-gene-related-peptide
CRPS	Chronic regional pain syndrome
CSF	Cerebrospinal fluid
ELISA	Enzyme-linked immunosorbent assay
FMS	Fibromyalgia syndrome
IL	Interleukin
NRS	Numerical rating scale
RA	Rheumatoid arthritis
TLR4	Toll-like receptor 4
TNF-α	Tumor necrosis factor α
TRP	Transient receptor potential channel
TRPV1	Vanilloid-Receptor 1 transient receptor potential channel
WBC	Whole-body-cryotherapy

## Appendix A. Cohort Characterization

The mean age was 59.33 years (±8.56), with an age range of 43 years, with a maximum age of 83 and a minimum age of 40. Among the patient population, 32 patients had the primary diagnosis of fibromyalgia syndrome (52.5%). Another 27 patients suffered from a chronic pain disorder (44.3%). One patient had ankylosing spondylarthritis and another had graft arthritis (3.3%) as their primary diagnosis (Table A1).

**Table A1:** Breakdown of the collective based on the main diagnoses and groups in absolute terms and percentages within the respective group.

Collective	FMS *n* =	Chronic Pain Disorder *n* =	Arthritis *n* =
Total *n* = 61	32 (52.5%)	27 (44.3%)	2 (3.3%)
Test group *n* = 37	21 (58.3%)	15 (41.7%)	1 (3%)
Control group *n* = 24	11 (44%)	12 (48%)	1 (8%)

Secondary diagnoses included depression in 30 patients (49.2%), degenerative bone or joint changes (including osteoarthritis of unspecified location) in 37 patients (60.7%), other rheumatic diseases in 26 patients (42.6%), concomitant neurological diseases in 11 patients (18%), and chronic headaches in 10 patients (16.4%).

Using the Hannover Functional Questionnaire, 36 patients provided a subjective assessment of their current limitations in daily life [33]. The average preserved functional capacity was 56% (±17%).

During the course of complex treatment, 36 of the subjects continued their existing medication, while 25 had their medication changed. 19 patients received additional medication or an increased dose of previously prescribed medication. One of these was classified as a biologic. For the remaining 6 patients, either the dose was reduced or an existing medication was discontinued.
